# Malaria Vaccine Development and How External Forces Shape It: An Overview

**DOI:** 10.3390/ijerph110706791

**Published:** 2014-06-30

**Authors:** Veronique Lorenz, Gabriele Karanis, Panagiotis Karanis

**Affiliations:** 1 Center of Anatomy, Medical School, University of Cologne, Cologne 50937, Germany; E-Mail: veronique.lorenz@web.de; 2 National Research Center for Protozoan Diseases, Obihiro University for Agriculture and Veterinary Medicine, Hokkaido 080-8555, Japan; E-Mail: gabrielekoydl@yahoo.de; 3 Centre for Biomedicine and Infectious Diseases (CBIF), Qinghai Academy for Veterinary Medicine and Animal Sciences, Qinghai University, Xining, Qinghai 810016, China

**Keywords:** malaria, malaria life cycle, malaria vaccine development

## Abstract

The aim of this paper is to analyse the current status and scientific value of malaria vaccine approaches and to provide a realistic prognosis for future developments. We systematically review previous approaches to malaria vaccination, address how vaccine efforts have developed, how this issue may be fixed, and how external forces shape vaccine development. Our analysis provides significant information on the various aspects and on the external factors that shape malaria vaccine development and reveal the importance of vaccine development in our society.

## 1. Introduction

According to the WHO, a child dies of malaria every 30 seconds. In 2008, the malaria burden comprised nearly 250 million cases of the disease, causing nearly one million deaths [1]. The economically disadvantaged are the most afflicted by the disease, resulting in a deterioration of economic status in highly endemic countries [2]. The decrease in per capita economic output is estimated at 50% compared to non-malarious countries [3]. Currently, half of the world’s population is at risk for malaria infection; infants and pregnant women are particularly vulnerable to severe morbidity and mortality due to malaria infection [2,4]. The greatest number of malaria cases occurs in sub-Saharan Africa; however, Asia, Latin America, the Middle East and parts of Europe are also afflicted [2].

## 2. Malaria Vaccine Development: Overview

### 2.1. Malaria—An Underestimated Danger in the West?

The massive incidence of malaria in the Third World makes the pressing need for further vaccine development undeniable. In the backdrop of malaria treatment, drug resistance and insecticides, appropriate means of disease control are currently lacking; the development of a vaccine remains the only tool for disease eradication.

Public interest in climate change is closely intertwined with concerns about the increased spread of tropical infectious diseases in Western countries. This development will heighten awareness and increase public demand for malaria vaccine research. 

Currently, the degree of knowledge regarding infectious disease risk factors, reflected by the prevention measures taken by travellers to malaria-endemic countries, appears dissatisfactory and calls for improvement via public information campaigns [5]. Among the pivotal factors that will amplify future confrontation with malaria are globalisation and migration [5].

In light of highly-developed socioeconomic structures, the malaria disease risk appears to be minimal in Western societies. Consequently, diagnostics and therapies quite likely would contribute to impeding the formation of a human reservoir [6]. Still, the lack of familiarity and expertise with the vast range of clinical malaria features may result in potentially lethal misdiagnoses in western medical systems. Important differential diagnoses, which occur with higher frequency, include gastroenteritis and influenza [7].

Equally problematic is the widespread lack of effort in collecting information for thorough case histories. A stay in a malaria-endemic area or a previous malaria infection with current relapse, in cases of *P. vivax* and *P. ovale*, may be overlooked.

An illustrative example of the misdiagnosis of malaria is the case of a 38 year-old Munich man who, upon his return from Kenya, displayed textbook symptoms of malaria, which doctors failed to recognise. After a diagnosis of influenza, no further diagnostic measures were performed and the patient expired [8].

This case helps broaden our understanding of the reasons for the considerable, approximately 3.9% malaria case fatality in Germany [9]. In the context of our highly developed health infrastructure, the impressive number of lethal cases confronts us with our inadequate knowledge of tropical diseases in general, and malaria specifically, that requires improvement [10].

### 2.2. Lessons Learnt

The history of malaria allows us to identify how humankind substantially increased the spread of malarial disease: the invasion of the mosquito’s habitat initiated the exposure of humans to vector-borne disease.

Equally important in the analysis of malaria history is our understanding of the major impact of social and political structures on disease endemicity [11]. For instance, the U.S. support of malarious countries during the Cold War depended on a country’s obedience to political U.S. leadership. This flawed arrangement strongly contributed to unsuccessful eradication attempts, as ineffective intervention methods strongly contributed to insecticide resistance [12].

The latest intervention programmes have proven efficacy. Nevertheless, we must cautiously reflect on the lesson learned with DDT that proved how attempts at unilateral disease eradication are prone to failure. Various scientific approaches to malaria eradication do not take into consideration the economic aspects of disease endemicity: malaria is a disease of the poor. For example, economic status, the use of insecticide-treated bed nets and children’s anti-malaria treatment are positively correlated [11]. 

The Global Malaria Strategy already recognised how a multidimensional approach to disease eradication also must include the general improvement of standards of living: “Malaria control is not the isolated concern of the health worker. It requires partnership of community members and the involvement of those involved in education, the environment in general, and water supply, sanitation and community development in particular. Malaria control must be an integral part of national health development and health concerns must be an integral part of national development programmes”. However, these rightly formulated ideas still await fulfilment [11].

The underlying rationale for the widespread focus on a scientific micro-cosmos requires thorough analysis. Firstly, Western interests in malaria medication and vaccine development may be attributed to egoistic intentions. Western travellers, who represent the most desirable target group on an economic basis, would directly benefit from a vaccine against pre-erythrocytic malaria stages.

Secondly, researchers have frequently and correctly been characterised as competitors getting lost in episodes of rivalry that hamper research through a lack of collaboration. One of the earliest documented examples of this misbehaviour in malaria research was between Grassi and Ross. 

Most importantly, we must focus on our goal: the development of an efficacious vaccine that is required to eradicate malaria. The example of smallpox vaccination and eradication provides this evidence. Nevertheless, the eradication of malaria, a vector-borne disease, calls for interventional processes, broadened from a medical basis to a socioeconomic context [13].

### 2.3. Why Have All Vaccine Approaches Remained Unsuccessful So Far?

Almost all scientific reviews about the currently most advanced vaccine candidate RTS,S come to a clear consensus: the formidable task of malaria disease eradication will not be accomplished with this vaccine.

Its main component is the sporozoites’ surface protein, the so-called circumsporozoite protein (CSP). The abbreviation RTS,S, includes an “R” for the B-cell repeat epitope of the CSP, a “T” for the T-cell epitope region of the CSP and an “S” for the hepatitis B surface antigen [14]. 

The hepatitis B surface antigen protein is grown in yeast and can spontaneously form virus-like particles with high immunogenicity. A fusion of CSP to the hepatitis surface antigen protein is performed to enhance the production of antibodies against CSP. To maintain the hepatitis B surface antigen’s ability to self-assemble, one part RTS is mixed with four parts hepatitis B surface antigen. A crucial step of vaccine engineering lies in the combination of these components with an adjuvant named AS02, which consists *inter alia* of lipids and an extract from the Chilean soapbark tree [13]. The typical standard aluminium adjuvant was discredited for application in a malaria vaccine, as it mainly evokes antibody responses. Due to the *Plasmodium’s* highly advanced immune evasion capabilities, a sole antibody response was regarded as insufficient, and the use of an adjuvant also supporting the T-cell response was favoured instead [15].

Despite these considerations, the lack of enduring protection remains a major disadvantage of this vaccine approach. Results of Phase III trials from 2013 displayed moderate results with 46% fewer cases of clinical malaria and a reduction in severe malaria by approximately 36% in infants aged 5 to 17 months, who were vaccinated once with RTS,S [16]. However, trials performed over a longer period showed a massive time-related decline in RTS,S’ efficacy. An efficacy of 16,8% was calculated for infants vaccinated three times after a follow-up of 4 years [17]. Pivotal for these disappointing trial outcomes, appears to be the brevity of antibody response [13]. The discouraging results with the precursor subunit vaccine, SPf66 (Serum *Plasmodium falciparum* version 66) had already indicated how the basis of the vaccine approach might be flawed [18]. 

In the late 1980s, Manuel E. Patarroyo, a Colombian, developed the vaccine SPf66 consisting of a polymer, which contained peptides from the surface of the merozoite (MSP-1 among others) and CSP. The first testing of SPf66 was performed in monkeys and then in humans. Trials from 1993 conducted in Brazil, Colombia, Ecuador and Venezuela with 41,000 people appeared to bode rather well, documenting an efficacy between 40% and 60%, although criticism was voiced concerning trial procedures. The trials had been performed without a control group and did not follow the gold standard of double blindness. The trials that followed, conducted with children in Tanzania and Thailand, also delivered disappointing data: very minimal data were collected and two years after vaccination, no protection against malaria infection was reported [19].

As research on the genome of *P. falciparum* demonstrated, it comprises approximately 5400 protein-coding genes with some only expressed at specific stages [20]. Disappointing results with a vaccine candidate that triggers antibodies only against a single surface protein therefore do not appear surprising.

Apart from its failure to trigger enduring strong immunity to malaria, the candidate RTS,S again revealed how extensively vaccine candidates are tested before their market release. Approximately 30 years will have elapsed from its first development to market release. In general, this constant and persistent concentration on one vaccination candidate bears the danger of neglecting other possibly sensible approaches to vaccination. However, a research programme that already includes 20 years of research, partly financed by a private company and improving the image of the latter, appears unlikely to be terminated without the prospect of a profitable outcome.

To guarantee the highest quality and safety of future vaccine candidates, we can anticipate that they will be subject to similar extensive and expensive trials, making the probability of the release of an efficacious malaria vaccine within the next 10 years unrealistic.

Other vaccine candidates display additional highly problematic flaws, making them prone to fail in solving the formidable task of malaria disease limitation. Proponents of an approach targeting the erythrocytic stages neglect the fact that *P. vivax* and *P. ovale* can develop so-called hypnozoites inside the liver and potentially cause a relapse of disease even after many years.

More importantly, the antigenic diversity of the parasite’s erythrocytic stages appears to primarily drive immune evasion. Finding a vaccine that successfully disrupts the life cycle at this point therefore appears to be elusive.

However, research on the erythrocytic stages of malaria has delivered significant scientific material. The evolution of erythrocytic cell defences that develop in response to malaria directs our attention to two significant phenomena: firstly, the human immune system has proven unable to combat the *Plasmodium* and has adapted to coexistence with the parasite. This elucidates how an imitation of natural responses may be a flawed approach to malaria vaccine development. The frequently described “clinical immunity” does not meet the criteria of the actual term “immunity”. Individuals do not avoid infection; instead, they suffer from repeated infections with malaria but experience fewer clinical symptoms. Nevertheless, they represent a human reservoir for the *Plasmodium.* Our goal must be to limit this human reservoir by vaccination to yield long-term reductions in the number of malaria transmissions. Secondly, the red cell defences are a reminder of the remarkably long history of malaria.

According to the author Paul W. Ewal, microbiologist René Dubos claimed in 1965, “*Given enough time a state of peaceful coexistence eventually becomes established between any host and parasite*” [21]. The extent to which the coexistence of, for instance, sickle-cell disease and malaria really is peaceful, remains to be discussed, as individuals with sickle-cell disease have a decreased life expectancy compared to healthy individuals [22]. Therefore, the proposal of host-directed drugs that imitate natural host defences appears to be problematic [23]. Bearing in mind the successful forms of malaria medication, the risks do not outweigh the costs of this form of malaria treatment. 

Unfortunately, the one natural host change that sufficiently prevents infection and is not accompanied by any health-impairing consequences for the host is the Duffy antigen null. Individuals with the Duffy antigen null are protected from *P. vivax* but not from *P. falciparum*. However, we might expect René Dubos’ prognosis also to come true for the youngest form of the *Plasmodium* in the future: with evolutionary changes that peacefully impede the *P. falciparum* erythrocyte interaction.

The erythrocytic stage as a point of intervention into the *Plasmodium’s* life cycle is clearly too late and therefore insufficient; the parasite’s capacity to hide inside human cells hinders a successful disruption of the life cycle. If the vaccine approach is targeted to the erythrocytic surface, the parasite can evade destruction through antigenic variation. Intracellular structures, therefore, seem to represent a more reasonable approach. Still problematic is the possibility of autoimmune responses due to structural similarities between human and parasite metabolism that impede the proposed approaches [24].

Secondly, and more importantly, this vaccine approach is not particularly attractive for Western travellers, who, although they represent only a fraction of the vaccine target group, majorly drive research efforts [25]. Research, after all, is predominantly performed in Western countries.

Vaccine approaches targeting the life cycle at an even later point face other hurdles. Approximately 60 years have elapsed since the first theories on transmission-blocking vaccination were presented on the basis of investigations with chickens vaccinated with avian sexual and asexual stage malaria parasites and were shown to develop a form of transmission-blocking immunity [26].

Among the first candidates suggested were the proteins Pfs 25 and Pfs 28 that are exclusively displayed on the surface of ookinetes and zygotes in the mosquito stage of infection [27]. Considered to be possibly problematic is their lack of natural occurrence in humans that impedes a boosting by natural infection and represents the cause of the protein’s low immunogenicity [28]. 

The analysis of malaria history reveals how malaria is a dynamic disease accompanied by mutations in the host and, as ongoing research on antigenic variation reveals, also in the *Plasmodium* itself [4]. It therefore appears highly doubtful that manipulation of the *Anopheles* mosquito or the sexual stages of the *Plasmodium* and the involved proteins required for the completion of the life cycle will lead to an enduring limitation of disease. Not only can an alteration of *Plasmodium* surface proteins be expected to hamper intervention attempts in mid-gut and salivary gland invasion, but it also seems that an altered virulence or even the adaption to a new vector is likely to bypass the interventional approaches targeting the vector (e.g., sterile insect technology). After all, the parasite has proven its magnificent adaption capacities in the past.

Finally, a vaccine approach that is often praised as a milestone, apparently demonstrating how sterile immunity can be reached, is that of whole-parasite vaccines. The first successful trials based on the theory of whole-parasite malaria vaccines were conducted in birds, which were partially immune against infection after immunisation with killed sporozoites or sporozoites inactivated with ultraviolet light [29]. 

Nussenzweig *et al.* (1967) chose a different mechanism for the attenuation of sporozoites and vaccinated mice with X-irradiated sporozoites of *P. berghei* [30]. The results revealed a more than 90% protection in mice to challenge with unattenuated sporozoites that persisted approximately 2 months [29].

Evaluations of this approach seem overly optimistic as the trials were in fact conducted with a very limited number of volunteers and ultimately failed to exhibit enduring immunity. Consequently, it seems unwise to rely on these results for future vaccine approaches.

There are additional major organisational hurdles that impede the idea of performing mass immunisation with X-irradiated mosquitoes infected with *P. falciparum.* Among other issues, the attenuated parasites would have to be stored frozen, which is an additional financial burden and is especially problematic in those tropical countries, which represent the main target group of malaria vaccination [31].

### 2.4. What Do We Expect of Future Malaria Vaccine Candidates?

Before analysing the various possibilities and approaches to malaria vaccination, a definition of our expectations for a malaria vaccine is required.

Currently, applied vaccines obtain a relatively high rate of efficacy, approximately 90% [32].

With regard to a potential malaria vaccine, many scientists agree that these high standards will not be met. It was frequently stated how sterile immunity elicited through a vaccine could not be expected due to the lack of real natural immunity to malaria. Indeed, scientific data only offers cases of the before-quoted clinical immunity that decreases disease burden [4]. The ambitious aim then could be to develop a vaccine candidate eliciting an immune response that is superior to the natural response to infection [33]. 

Other scientists, though, are less optimistic and, with regard to many years of limited success, come to the conclusion that even a rather poorly performing vaccine such as RTS,S could accomplish a massive reduction of malaria lethality [13].

Even so, from an economic point of view, this approach finds less approval. A vaccine with low efficacy is much less cost effective in contrast to scaling up malaria treatment and investing in vector control programmes such as insecticide-treated bed nets [34]. Related to these economic factors, it has even been proposed to extend disease control with interventional methods and to terminate the thus far unsuccessful malaria vaccine research. Surely this must be regarded as a rather naive proposal: the major hurdle of resistance against malaria medication and insecticides remains unscathed and can be expected to occur with every new medication or insecticide. A vaccine against malaria is still required; however, it will have to be cost effective and elicit enduring and strong immune responses [20,34]. Otherwise, the useful resources that could be applied for more sensible vaccine approaches and effective interventional methods will be lost. 

At this point, we must define vaccine efficacy and clearly state that efficacy would be characterised by a parasitaemia of individuals living in malaria-endemic areas of approximately zero. This has a deeper rationale due to the fact that the human reservoir that distinguishes malaria endemicity from so many other infectious diseases must be combated to achieve malaria eradication.

A further significant aspect of vaccine development that should be considered is the target group. For economic reasons, it would be middle-aged Western travellers, but from a sensible point of view, it would be young infants <1 year of age and pregnant women in whom the vaccine candidate should prove efficacy.

The illustrative numbers are as follows: in Sub-Saharan Africa, malaria infection causes approximately 400,000 cases of severe maternal anaemia and up to 200,000 infant deaths a year [35]. Therefore, it is lamentable that research in areas such as the proposed vaccine for mothers sees so little progress in comparison to candidates such as RTS,S. Nonetheless, RTS,S hopefully will contribute to a reduction in child mortality from malaria, as it may give small infants additional time to develop a form of clinical immunity. However, it is essential to insist on the long-term goal of the global malaria vaccine community and continue along this arduous path that hopefully will lead us to a malaria vaccine with 80% efficacy by 2025 [36].

### 2.5. What Kind of Research Do We Need for the Successful Development of a Malaria Vaccine?

The review of malaria research findings and even the latest approaches to malaria vaccination intriguingly reveal how incomplete the scientific knowledge of the molecular processes of the *Plasmodium’s* life cycle is. Especially, the initiation of infection; the skin stage represents a stage of the life cycle that awaits further detailed investigation [37]. Looking back on many years of malaria research, it stands out that establishing a deepened comprehension of the actual life cycle may be the crucial requisite before an efficacious vaccine candidate can be developed.

It appears striking why so few scientists have thus far insisted on basic research before initiating research on a specific vaccine candidate. The answer may again be rooted in a hunt for personal prestige, which is surely not guaranteed for those standing at the beginning of a long research path. Scientific acknowledgement or even a Nobel Prize presumably awaits the scientist eventually developing the long-desired vaccine; therefore, it appears much more interesting for scientists to directly participate in a project for a specific vaccine candidate. Clearly, the ongoing participation in clinical trials of certain vaccine candidates also offers greater possibilities for publication than long-winded basic research that may lead to inconclusive results.

The importance of basic malaria research has a profound reason that separates the hunt for a malaria vaccine from all the other past vaccine developments: malaria is a parasitic disease, and currently, no human vaccine against any parasitic disease exists [38]. Therefore, the argument of the very successful smallpox vaccine having been developed with little knowledge of host immunology can be easily refuted, as the sheer complexity of the *Plasmodium* parasite’s genome in comparison to the smallpox virus justifies our call for basic research [19].

Parasitism is defined by the parasite being able to live at the expense of the host; this is an unpalatable fact that clearly divides malaria from other infectious diseases [39,40]. A parasitic characteristic of outstanding significance is its ability to evade the immune system successfully. There are numerous immune evasion mechanisms within the life cycle. 

Trials in mice suggested that the induction of immunotolerance starts early within the life cycle. The presence of sporozoites within the skin was demonstrated to increase the mobility of skin Tregs; furthermore, a lower proliferation of skin-migrated CD4-cells could be noted [41,42].

Additionally, it stands out that each stage of the parasite’s life cycle is characterised by certain antigens making the number of antigens expressed altogether so broad that it seems unlikely that a vaccine intervening at one certain point will successfully disrupt the entire life cycle. The vaccine would have to exhibit an unrealistic 100% efficacy to prevent the deployment of subsequent stages of the life cycle, which can again rapidly reproduce [11].

Thus far, scientists have failed to tackle the challenge of parasitic immune evasion; even so, specific stages of the life cycle appear more promising than those we have focused on so far. We therefore propose an intervention that is efficacious before the parasite’s immune evasion: an intervention targeting the skin.

### 2.6. The Skin as a Malaria Vaccination Target

Considering the density of the Langerhans cells and DCs inside the skin, which is higher than inside the muscle, this first barrier of our immune system may halt the parasite’s invasion altogether [41,42]. The first immune response is based on the Langerhans cells internalising antigens and inducing a T-cell response in the draining lymph node [43]. By contrast, the majority of currently available vaccines give rise to humoral responses [41]. Nevertheless, the analysis of the *Plasmodium*’s life cycle clearly indicates how only a cellular immune response can combat the *Plasmodium* during the skin stage. A humoral response is unlikely to be competent for terminating the sporozoites’ migration process through the dermis, which is based on cell traversal [44].

The likely solution of transdermal vaccinations is also appealing for the following reasons: enhanced compliance and a decreased problem of safe syringe disposal, which are undoubtedly strong advantages [43]. 

In combination with the novel application route of transdermal vaccination, we can expect new vaccine compositions, especially DNA vaccination, to burst further into the limelight. 

Veterinary medicine creates a role model pathway that will expectedly be followed by human medicine. Two DNA vaccines and therapies that were both released in 2007 must be especially emphasized: the canine melanoma vaccine and GHRH (growth hormone releasing hormone), which is applied in swine. The two vaccines target very different problems; the melanoma vaccine is a form of tumour therapy. In contrast, GHRH is a preventive vaccine with decreased perinatal mortality and morbidity [45].

The DNA vaccination is based on the following construction: a plasmid, which is a circular piece of bacterial DNA, genetically modified to produce proteins of a certain microbe or virus, is inserted into a human cell. The host cell expresses proteins on the basis of this new DNA and the immune system recognises these as foreign [45]. Most DNA vaccines that are subjects of current research programmes do not integrate into the host’s cellular DNA; they only enter the nucleus [46].

The canine melanoma vaccine serves as an illustrative example to deepen insight into the process of DNA vaccination. The vaccination results in an expression of the human tyrosinase gene, which differs from canine’s tyrosinases and thus evokes an immune response in dogs. Importantly, the human tyrosinase resembles the canine’s tyrosinase to an extent high enough to induce down-regulation of the canine’s tyrosinase as well. This serendipitous fact efficiently inhibits tumor growth [47].

There is wide agreement that one of the major immunological advantages of DNA vaccination is the activation of CTL-cells. CTL-cells require antigen presentation through MHC I, which is achieved either through direct transfection of the APCs by the plasmid vaccine or through cross-presentation. The term of cross-presentation describes the APC’s ability to ingest infected necrotic or apoptotic cells and present the exogenous antigen via MHC I [46].

A further pivotal argument in favor of DNA vaccination is economic. An attractive property of DNA vaccination is its inexpensive and pure manufacturability, which does not encounter the various production hurdles of other vaccine approaches [47].

Currently, efforts are ongoing to face a problematic weakness of DNA vaccines: their rather low immunogenicity in humans [46]. The DNA vaccination advancements made in veterinary medicine appear to be significant developments pioneering a future vaccine for humans. DNA vaccination is one of the novel approaches for developing a new generation of vaccines against malaria [48].

### 2.7. How Money and Research Funding Drive the Malaria Vaccine Efforts

Clearly, the investment in certain research areas is strongly influential to the acceleration or deceleration of research. The EU support provides an illustrative example. In 2009, basic malaria research was supported with €15.9 million. Research was coordinated by the French Pasteur Institute and concentrated on parasite genetics, cell biology and metabolism, pathogenesis, immunology and the mosquito vector. Malaria vaccine development was supported with only a little less: €13.5 million [49]. In 2006, resources were invested in 16 candidates that were in clinical development [36]. The value of these investments is controversial, especially with regard to the poor performance of the most advanced vaccine candidate; it offers a protection rate of only 30% [13]. Critics disapprove of the cost-intensive testing of ineffective vaccine candidates and demand that the generation of superior vaccine candidates be based on basic research [50].

Scientists appear to be pressured by the investing pharmaceutical companies and therefore tend to offer an overly optimistic hope of reaching the aim of a successful vaccination soon. An illustrative example is scientist William Trager, who in the 1970s stated in an unofficial context, “*We must promise a vaccine is on the horizon or else research funding will quickly dry up*” [39]. Evidence accumulates to suggest that this form of pressuring may in fact have slowed the research pathway and triggered a generation of various vaccine candidates that lacked a basic research background. 

Generally, we are faced with the hurdle that vaccine development represents a scientific branch that includes long-winded and expensive regulatory processes and may even result in a rejection of the tested vaccine candidate. Given the cost and risks of the manufacturing process, very few companies show interest in malaria vaccine research and prefer to focus on research areas that are surely profitable [1].

These limiting economic factors massively hinder vaccine development and have contributed to the status quo with RTS,S, which has undergone cost-intensive development and will soon be released to the market, even if its efficacy remains highly doubtful.

Overall, the vaccine market was predicted to flourish in the future; however, this prognosis may be correct only for vaccine candidates for illnesses that are of concern in western societies, such as vaccines against cancer.

### 2.8. The Role of the State in Malaria Vaccine Development

The financial and scientific contributions to vaccine development should be accelerated through broader governmental support of vaccine industries. Furthermore, the extent of regulatory mechanisms of vaccine development may have to be reviewed to accelerate the development and market release of vaccine candidates. Especially regarding the demand for vaccine candidates that cause little to no adverse events, there appear to be exaggerated safety concerns, as the benefit of hundreds of successful vaccinations preventing serious diseases outweighs the cost of a very rare adverse event [51].

Additional support for vaccine development for diseases with predominant incidence in third-world countries is obviously favourable. The cost effectiveness of a sufficient vaccine will outweigh the massive costs of other interventional measures (e.g., bed nets, insecticide spraying) and malaria medications that currently are being utilised for disease limitation. Equally important is the economic stabilization of third-world countries, which also is of interest to western democracies. Endemic malaria infections and poverty are correlated and further linked to overall instability and a weak economy in affected countries [3]. Clearly, the community of economically successful countries should therefore feel an obligation to stabilise these countries that currently represent potentially threatening reservoirs of disease but also represent potential trade partners in the future.

Various measures show the potential to further enhance the development of life-saving vaccines, such as limited taxation for companies researching life-saving rather than profitable products and heightened support for vaccine manufacturers that face scientifically ill-founded trials raised by anti-vaccine movements [51].

Lastly, western states should have a vital interest in raising the awareness of parasitic diseases in the medical community. As previously discussed, malaria remains an underestimated danger in western countries. This is not only unfavourable and potentially dangerous in the event of an actual infection, but the lack of knowledge concerning malaria is, with regard to the current and future global impact of the disease, simply inadequate. Especially among medical students and the younger generation of doctors, most of whom have not been confronted with malaria patients, disease awareness must be increased through improved integration of the topic into their curriculum.

### 2.9. The Influence of the Public on Malaria Research and Control

The history of malaria and vaccination in general has shed light on the fact that public opinion massively influences the state’s application of certain forms of disease control and compliance within the population.

Clearly, the degree of knowledge about infectious diseases requires improvement and can be assumed to be directly correlated with population compliance with vaccination. The example of the polio vaccine supported by the campaign the March of Dimes in the 1940s and 1950s in the U.S. clearly illustrates how well-informed individuals who were personally affected by polio proved to be a great resource for encouraging public compliance with vaccine uptake [51].

Information politics must be forwarded before infectious diseases that are neglected by younger generations, such as measles, find an upsurge.

Equally important is that the western public can indirectly drive developments for third-world countries if the ongoing discussion on climate change brings the issue of infectious disease control further into the limelight.

### 2.10. What Can We Expect in the Near Future with Regard to Malaria Vaccine Development?

Generally, we can expect an extension of disease control based on vaccination in the future, with an increasing number of non-infectious diseases being targeted by vaccination. Concerning the unique effect of vaccination on general life expectancy so far, this must be considered a positive development [50].

Additionally, in contradiction to pessimistic evaluations on the unfavourable market of vaccine development, we might still hope for public-private partnerships to further enhance the development of the vaccine market, especially for vaccines that mainly target Third-World populations [50].

Notably, the vaccine market will not only broaden to include non-infectious diseases due to the increasing impact of chronic diseases, but we also can expect new techniques, including DNA vaccination, to open up doors for the thus far unsuccessful vaccination attempts.

However optimistic one might be, we must make a realistic prognosis and state that a highly effective malaria vaccine within the next ten years appears to be unrealistic based on the scientific status quo.

## 3. Conclusions

Due to the lack of an efficacious vaccine approach to date, vector control programmes, including insecticide-treated bed nets, artemisinin combination therapies and insecticides, have been applied. These measures are considered with the support of approximately $1 billion dollars and have been estimated to decrease the malaria disease burden by 90% (measured were cases of death and admissions to hospitals). Critical reports predict RTS,S, the currently most advanced vaccine candidate, would only improve these numbers by a meagre rate of 3%. Therefore, conclusions were drawn as to which only partially effective vaccination will yield poorer results than other malaria control measures.

Currently applied vaccines display a relatively high rate of efficacy, approximately 90%. With regard to a potential malaria vaccine, however, parts of the scientific community are in agreement that these high standards will not be met. Other scientists, though, conclude that even a rather ill performing vaccine such as RTS,S could accomplish a massive reduction of malaria lethality. Hopefully, RTS,S will particularly contribute to a reduction in child mortality, as it may provide infants with more time to develop a form of clinical immunity. Nonetheless, almost all the scientific reviews about the currently most advanced vaccine candidate RTS,S come to a clear consensus; the formidable task of malaria disease eradication will not be accomplished with this vaccine. As previously stated, the prospect of a lack of enduring protection remains a major disadvantage of this vaccine approach. The disappointing results with the precursor subunit vaccine SPf66 have already indicated how the basis of the vaccine approach might be flawed.

A generally reported problem concerning subunit vaccines and recombinant proteins is rooted in the fact that artificially produced proteins only partially resemble the conformation of the parasite protein. This aspect may constitute the crucial weakness of this vaccine development and contribute to the inadequate immune response.

Vaccine approaches targeting the skin stage of infection, as well as other approaches targeting pre-liver stages, are united by an essential advantage: they take into consideration that two species of the *Plasmodium* (*P. vivax* and *P. ovale*) induce the production of so-called hypnozoites in the liver. Other vaccine approaches intending to intervene at later stages of the *Plasmodium’s* life cycle neglect the fact that the hypnozoites, even after years of physical health, represent a serious threat, capable of causing a relapse of disease. The only recently discovered skin stage of infection requires further elaborated research before a promising vaccine candidate may be expected.

As malaria has been a disease affecting humankind for a very long time, it has been investigated to have had substantial influence on human genetics. Haldane pioneered hypothesising the possibility of natural selection, pressured by infectious diseases. Critically evaluated on the basis of scientific record and research, Haldane’s early “malaria hypothesis” could indeed be proven to be correct. The analysis of distinctive natural changes in erythrocytes might be highly advantageous for the development of a vaccine. For instance, the temporary induction of G6PD- (glucose-6-phosphate dehydrogenase) deficiency was proposed to mimic the natural immunity of individuals with this gene mutation against malaria. Furthermore, the evolution of erythrocytic mutations demonstrates the ineffectiveness of our natural immune response against malaria and reveals how vaccination may have to target the induction of a form of non-natural immunity.

Apart from genetic changes in humans, the parasite’s genetics also alter the course of an infection as a means of immune evasion, this is called antigenic variation. Ongoing research in the field of antigenic variation may greatly contribute to successful vaccine development: increased genetic knowledge concerning antigenic variation as well as our inter-individual different immune reactions and susceptibility to the *Plasmodium* may enable us to develop more effective vaccine approaches. Scientific research in endemic areas revealed how antibody responses dominantly contribute to the mammalian host’s defence mechanism against the blood stages of infection. The antibody analysis proved how they are not distinctly directed against any single antigen but operate broadly. Current observations imply the synergistic significance of cellular immunity and humoral immunity but demand our cautious review.

Additionally, malaria infections may even be influenced by co-infections. The immune reaction to malaria as well as the loss of immunity to malaria after leaving endemic areas requires further elucidation. A broadened knowledge of this natural human response to the *Plasmodium* represents a significant basis for future attempts of inducing non-natural immunity by vaccination. 

Vaccine development specific to malarial disease in pregnant women, who are especially susceptible to malaria infection, appears to be a very reasonable approach that calls for advanced support in the future. The restricted target group, however, lowers the likelihood of noteworthy financial support through western countries.

Another field of vaccine approaches is represented by transmission-blocking vaccines. Sixty years have elapsed since the first theories on transmission-blocking vaccination were presented on the basis of investigations with chickens that were vaccinated with avian sexual and asexual stage malaria parasites and were shown to develop a form of transmission-blocking immunity. Transmission-blocking vaccines have been predicted to only extend disease control if applied in combination with other interventional methods, such as exposure prophylaxis and vector control. Alternatively, their usage in combination with poorly efficacious pre-erythrocytic vaccines or blood-stage vaccines might be proposed.

With regard to new approaches in vaccine application, we can expect plastid transformation to be of huge significance in the future, as it offers the possibility of foreign gene expression in high plants. The efficiency of plastid transformation requires the improvement of a routine application of plastid transformation that so far can only be documented for tobacco. 

As long as no effective vaccine candidate is discovered, vector control remains a significant and highly political topic that always represents, whether conducted biologically or chemically, a massive intervention into the environment with possible harm to animals and humans. Because resistance to any form of chemical vector eradication can occur and sterile insect technique cannot offer broad protection, vector control as a form of malaria disease control must be considered a temporary measure that is required until the development of a vaccine is finally achieved.

Apart from the scientific micro-cosmos, we must look at the political and economic dimensions of malaria disease control; a successful malaria vaccination programme is strongly dependent on the financial interests of the pharmaceutical companies and efficient vaccine distribution mechanisms. After all, an effective vaccine that does not reach its target group for logistic reasons can prove no use.

Moreover, it remains a major future task to improve public understanding about vaccination and the massive impact of infectious diseases. Particularly in schools and kindergartens, enhanced information politics have proven to be highly effective and require further extension. Population compliance is of great importance: herd immunity with a vaccine uptake of 80%–90% is required for disease eradication.

While the research platform is driven forward, it must however, be acknowledged that malaria is a dynamic disease: simian malaria requires our consideration. Theories have been constructed as to the possible causes of this transmission from monkey to human. Reports on human infections with *P. knowlesi* have led scientists to the suggestion that *P. knowlesi* may be the 5th human malaria parasite. These parasite dynamics must be regarded in the development of new vaccine approaches. Vaccination with a certain protein could, for example, only offer protection against certain *Plasmodium* species.

Looking back on many years of malaria research, we can conclude that establishing a deepened comprehension of the actual life-cycle might be the crucial requisite before an efficacious vaccine candidate can be developed. The currently most advanced vaccine candidates lack this background and will therefore not serve the long-term goal of malaria eradication.

The public interest in climate change is closely interlinked with concerns about the increased spreading of tropical infectious diseases in Western countries. This development will presumably increase awareness and support the scientific community of malaria vaccine research. 
